# Austin District Medical Society—18th Quarterly Meeting

**Published:** 1892-04

**Authors:** T. J. Bennett

**Affiliations:** Secretary


### AUSTIN DISTRICT MEDICAL SOCIETY.

The eighteenth quarterly meeting of the Austin District Medical Society was held in Medical Hall, Austin, March 24th, at which there was a good attendance of the physicians of the district.

Dr. G. W. Christian in the chair.

Dr. F. S. White, of Austin, the new lunatic asylum superintendent, and Dr. W. R. Barton, of Salado, were admitted to membership.

The following papers were read and discussed:

"Diphtheria and Croup," by Dr. W. P. Fleming, of Georgetown; "Obscure Mental Derangements" (published in this issue of the TEXAS SANITARIAN), by Dr. F. S. White, of Austin; "Backward Displacements of the Uterus," by Dr. W. J. Mathews, of Austin; "McLaughlin's Physical Theory Criticised," by Dr. A. N. Denton, of Austin.

The following delegates were elected to represent the Society at the Tyler meeting of the State Medical Association, April 26th:

Dr. Sam Cunningham, Elgin.
" T. D. Wooten, Austin.
" G. W. Christian, Burnet.
" A. Garwood, New Braunfels.
" W. T. Richmond, Manor.
" J. W. Hamilton, Lampasas.
" J. W. Carhart, Lampasas.
" F. R. Martin, Kyle.
" Fannie Leake, Austin.
" Seth M. Morris, Galveston.
" J. E. Thompson, Galveston.
" B. E. Hadra, Galveston.
" W. R. Barton, Salado.
" E. Clark, Lockhart.
" R. Atkinson, San Marcos.

Dr. F. S. White, Austin.
" J. F. Dean, Hornsby.
" T. J. Bennett, Austin.

On motion the Secretary was instructed to issue certificates to alternates in case any of the above regular delegates could not attend the Tyler meeting.

The next regular meeting of the Society will be held in Medical Hall, Austin, Thursday, June 23, 1892.

T. J. BENNETT, Secretary.

